# Hypersensitivity to Thromboxane Receptor Mediated Cerebral Vasomotion and CBF Oscillations during Acute NO-Deficiency in Rats

**DOI:** 10.1371/journal.pone.0014477

**Published:** 2010-12-31

**Authors:** Béla Horváth, Gábor Lenzsér, Balázs Benyó, Tamás Németh, Rita Benkő, András Iring, Péter Hermán, Katalin Komjáti, Zsombor Lacza, Péter Sándor, Zoltán Benyó

**Affiliations:** 1 Institute of Human Physiology and Clinical Experimental Research, Semmelweis University, Budapest, Hungary; 2 National Institute on Alcohol Abuse and Alcoholism, National Institutes of Health, Bethesda, Maryland, United States of America; 3 Department of Neurosurgery, University of Pécs, Pécs, Hungary; 4 Department of Control Engineering and Information Technology, Budapest University of Technology and Economics, Budapest, Hungary; 5 Department of Diagnostic Radiology, Magnetic Resonance Research Center, Yale University, New Haven, Connecticut, United States of America; Julius-Maximilians-Universität Würzburg, Germany

## Abstract

**Background:**

Low frequency (4–12 cpm) spontaneous fluctuations of the cerebrovascular tone (vasomotion) and oscillations of the cerebral blood flow (CBF) have been reported in diseases associated with endothelial dysfunction. Since endothelium-derived nitric oxide (NO) suppresses constitutively the release and vascular effects of thromboxane A_2_ (TXA_2_), NO-deficiency is often associated with activation of thromboxane receptors (TP). In the present study we hypothesized that in the absence of NO, overactivation of the TP-receptor mediated cerebrovascular signaling pathway contributes to the development of vasomotion and CBF oscillations.

**Methodology/Principal Findings:**

Effects of pharmacological modulation of TP-receptor activation and its downstream signaling pathway have been investigated on CBF oscillations (measured by laser-Doppler flowmetry in anesthetized rats) and vasomotion (measured by isometric tension recording in isolated rat middle cerebral arteries, MCAs) both under physiological conditions and after acute inhibition of NO synthesis. Administration of the TP-receptor agonist U-46619 (1 µg/kg iv.) to control animals failed to induce any changes of the systemic or cerebral circulatory parameters. Inhibition of the NO synthesis by nitro-L-arginine methyl esther (L-NAME, 100 mg/kg iv.) resulted in increased mean arterial blood pressure and a decreased CBF accompanied by appearance of CBF-oscillations with a dominant frequency of 148±2 mHz. U-46619 significantly augmented the CBF-oscillations induced by L-NAME while inhibition of endogenous TXA_2_ synthesis by ozagrel (10 mg/kg iv.) attenuated it. In isolated MCAs U-46619 in a concentration of 100 nM, which induced weak and stable contraction under physiological conditions, evoked sustained vasomotion in the absence of NO, which effect could be completely reversed by inhibition of Rho-kinase by 10 µM Y-27632.

**Conclusion/Significance:**

These results suggest that hypersensitivity of the TP-receptor – Rho-kinase signaling pathway contributes to the development of low frequency cerebral vasomotion which may propagate to vasospasm in pathophysiological states associated with NO-deficiency.

## Introduction

Low frequency (4–12 cpm) fluctuations of the cerebral oxygen availability in mammals were reported for the first time by Davis and Bronk in 1957 [1] and confirmed later in primates including humans (for review see: [2]). These fluctuations are termed “spontaneous” to indicate their independence of cardiac or respiratory cycles. In 1981 Dóra and Kovách [3] observed similar oscillations in cerebrocortical NADH fluorescence, which lagged by 2 sec behind the cortical vascular volume suggesting that the vascular event initiated the metabolic one. The concept that the low frequency metabolic oscillations of the brain are induced primarily by vascular changes and consequent variations of the cerebral blood flow (CBF) is also supported by observations on isolated cerebral vessels showing vasomotion in vitro [4]–[5]. Renewed interest in CBF oscillations was generated by human studies using laser-Doppler flowmetry [6]–[7], transcranial Doppler sonography [8], functional MRI [9] and near infrared spectroscopy [10].

Increased vasomotional activity, reflected by enhanced CBF oscillations has been reported in subarachnoid hemorrhage (SAH) before the onset of cerebral vasospasm and during the recovery from it [11]. This may indicate that overactivation of the mechanism(s) responsible for vasomotion may also participate in the pathogenesis of spastic vascular smooth muscle contractions and, consequently, increased vasomotional activity may predict the development of acute vasospasm. Contrary to this process, vasodilation (induced by hypercapnia or volatile anesthetics) promptly and reversibly suspends low frequency CBF oscillations [12]–[13] and the cerebrovascular endothelium also suppresses vasomotion by releasing nitric oxide (NO) [2]. In accordance, enhanced CBF oscillations have been reported in pathophysiological states associated with decreased bioavailability of NO, such as SAH [11], traumatic brain injury [14] and hypertension [15].

Previous studies on isolated cerebral arteries implicated the thromboxane pathway in the changes of vascular functions following NO synthase (NOS) inhibition [5], [16]–[17]. This interaction is particularly interesting in the light of recent observations indicating that in NO-deficiency other prostanoids (e.g. prostacyclin) may also induce vascular smooth muscle effects via activation of the thromboxane (TP) receptor [18] and cerebrovascular expression of TP-receptors is enhanced after SAH [19]. In the present study we hypothesized that in the absence of NO hypersensitivity of the cerebrovascular TP-receptor mediated signaling pathway contributes to the enhanced vasomotion and blood flow oscillations in the cerebral cortex.

## Methods

The experiments were performed in adult male Wistar rats (300–400 g) according to the guidelines of the Hungarian Law of Animal Protection (243/1988) and all procedures were approved by the Semmelweis University Committee on the Ethical Use of Experimental Animals (590/99 Rh). The animals were anesthetized with urethane (1.5 g/kg intraperitoneally), the depth of anesthesia was regularly controlled during the experiments by checking the corneal or plantar nociception reflex and additional urethane was administered intravenously (iv.) as necessary. The animals were spontaneously breathing through an intra-tracheal cannula. Catheters were inserted into both femoral arteries (for systemic arterial blood pressure measurement and for blood sampling) and into the left femoral vein (for drug administration). Body temperature was kept constant between 36–38°C with a controlled heating pad.

Systemic arterial pressure was recorded continuously on a polygraph (Model 7E, Grass, Quincy, MA, USA). Measurement of cerebrocortical blood flow (CoBF) has been performed by laser-Doppler (LD) flowmetry as described in detail elsewhere [20]. The head of the animals was fixed in a stereotaxic head holder with the nose 5 mm down from the interaural line. The skull of the parietal region was exposed and the bone was thinned over the parietal cortex on both sides with a microdrill, so that the lamina interna of the skull remained intact. Two LD probes were placed above the thinned skull at a 12°-angle to the vertical to provide an optimal view of the cortex (4 mm caudal from bregma, 5 mm lateral from midline). CoBF was measured with a two-channel blood flow monitor (MBF3D, Moor Instruments, UK) and was recorded continuously. The LD monitor was calibrated before each individual experiment with a constant movement latex emulsion. The laser light was in the infrared range (780 nm) and penetrated about 1 mm into the brain covering approximately 7 mm^2^ of the parietal region, so that the data acquired mostly represented the characteristics of the blood flow in the parietal cortex [20].

Animals were randomly assigned to four *in vivo* experimental groups. In the control Group I. systemic and cerebral circulatory parameters, as well as blood gas and acid-base values were determined before as well as for 75 minutes after an iv. bolus injection of 1 ml/kg vehicle (saline). Thereafter the animals received the thromboxane receptor agonist U-46619 in a dose of 1 µg/kg iv., which in preliminary experiments was below the threshold of inducing any systemic or cerebral circulatory changes. Groups IIa., IIb. and IIc. received intravenously first N^G^-nitro-L-arginine methyl ester (L-NAME) in a dose of 100 mg/kg for the inhibition of NO synthesis and 75 minutes later 1 µg/kg U-46619 (Group IIa.), saline (Group IIb.) or 10 mg/kg of the thromboxane synthase inhibitor ozagrel (Group IIc.). Previous studies have verified that L-NAME and ozagrel in the doses used in our present study effectively inhibit the activity of cerebral NO synthase and thromboxane synthase, respectively [21], [22]. The final measurements were performed in all experimental groups 50 minutes after the administration of U-46619, saline or ozagrel.

The *in vitro* experiments were performed in middle cerebral arteries (MCAs) supplying the parietal cortex, the site of the *in vivo* CoBF measurements. MCA segments were prepared from adult male Wistar rats and studied in a conventional myograph system (610M, Danish Myo Technology A/S, Aarhus, Denmark) as described previously [5], [23]. First, each segment was exposed to 124 mmol/L K^+^ Krebs solution to elicit a reference contraction. After a 30-minute resting period, the functional integrity of the endothelium was tested by application of bradykinin (0.01 to 10 µM) after precontraction induced by 100 µM UTP. Segments that did not exhibit at least 20% relaxation of the precontraction were considered to have damaged endothelium and were excluded from the study. After a 30-minute resting period, during which the baths were washed several times, the vessels received either 100 µM L-NAME in order to block NO synthesis or saline, the vehicle of L-NAME. Fifteen minutes later the effects of 100 nM U-46619 or 10 nM endothelin-1 (ET-1) were determined on the vascular tension both in intact and NO synthase blocked vessels. The role of Rho – Rho-kinase signaling pathway was tested by administration of 10 µM Y-27632, a specific Rho-kinase inhibitor [24], to NO synthase blocked vessels showing stable vasomotion after administration of U-46619 or ET-1. In additional control experiments intact MCA segments were precontracted with 25 mmol/L K^+^ Krebs prior to administration of 100 nM U-46619.

The Discrete Fourier transform (spectrum) of the time series obtained in vivo (CoBF) or in vitro (vascular tone) was calculated by Fast Fourier Algorithm (FFT) [25]. The calculations were executed in the Matlab environment which uses an adaptive version of the FFT, called FFTW [26]. The DC (zero frequency) component was eliminated from the spectrum by subtracting the mean value of time series from the samples and this way generating zero-mean time series. In order to identify the largest frequency component of the spectrum the region of interest was gated by an appropriate frequency window.

Since CoBF oscillations could also be induced by simultaneous changes of the blood pressure, we have performed the spectral analysis of the blood pressure recordings with the same method as described above. However, there was no difference in this parameter either between or within the experimental groups.

All chemicals were obtained from Sigma-Aldrich (St. Louis, MO, USA). Values are presented as mean ± SEM; n represents the number of experiments. Statistical analysis was performed using repeated measures ANOVA followed by a Tukey post-hoc test. A P value of less than 0.05 was considered to be statistically significant.

## Results

Baseline physiological parameters were within the normal range in all *in vivo* experimental groups (Table 1.). Neither iv. administration of saline nor that of the TP-receptor agonist U-46619 in a dose of 1 µg/kg induced any significant changes in acid-base, blood gas or systemic circulatory parameters in the control Group I. (data not shown). Furthermore, neither the average CoBF nor its Fourier spectrum changed after the administration of saline or U-46619 in this experimental group (data not shown). These observations confirmed that 1 µg/kg U-46619 has no significant effect on the systemic and cerebrocortical circulation under physiological conditions.

**Table 1:** Baseline physiological parameters in the different in vivo experimental groups.

Mesured Variable	Experimental Group			
	I.	IIa.	IIb.	IIc.
**Mean Arterial Pressure (mmHg)**	97.9±2.4	100.0±2.9	97.7±3.5	107.7±4.0
**Heart Rate (bpm)**	410±9	408±15	413±8	435±20
**PaCO_2_ (mmHg)**	41.0±2.6	43.7±1.5	38.2±1.6	40.0±2.0
**O_2_ Sat (%)**	96.5±0.5	96.5±0.5	96.6±0.3	96.2±0.3
**pH**	7.34±0.01	7.32±0.02	7.38±0.01	7.37±0.01
**Standard Base Excess (mmol/l)**	−3.7±1.1	−2.2±0.7	−2.6±0.4	−1.7±1.1

Values are mean ± SEM (n = 7, 10, 6 and 7 in Groups I, IIa., IIb. and IIc., respectively). No significant difference was found between the experimental groups.

**Figure 1:**
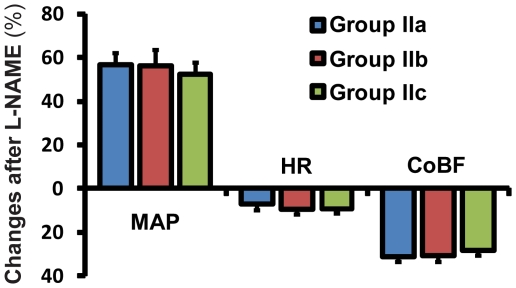
NO synthase blockade increases mean arterial pressure (MAP), while decreases heart rate (HR) and cerebrocortical blood flow (CoBF). Mean ± SEM percentual changes are shown after NO synthase inhibition by 100 mg/kg of L-NAME, compared to the steady state pre-injection values presented on Table 1 (n = 10, 6 and 7 in the case of MAP and HR, and n = 20, 12 and 14 in the case of CoBF in Groups IIa., IIb. and IIc., respectively).

**Figure 2:**
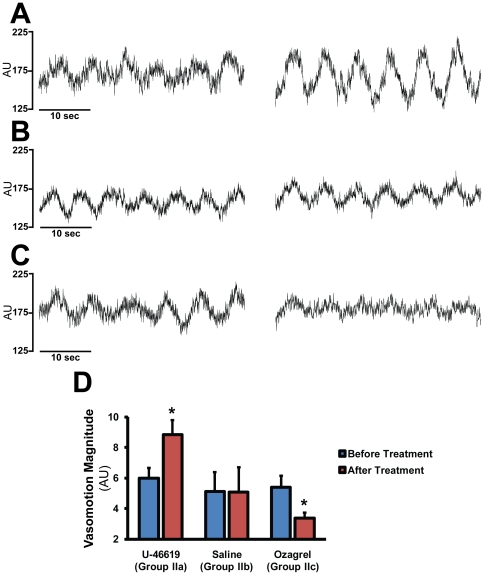
Weak activation of thromboxane receptors aggravates while inhibition of TXA_2_ synthesis attenuates CoBF oscillations developed in the absence of NO. **A–C**: Original recordings of the cerebrocortical laser-Doppler flux *in vivo* before (left panels) and after (right panels) administration of the TP-receptor agonist U-46619 (A), the thromboxane synthase inhibitor ozagrel (C) or their vehicle (saline) (B) in rats pretreated by the NO synthase inhibitor L-NAME. **D**: Quantitative analysis of slow wave oscillations with discrete Fourier transformation. The peak magnitudes of the power spectra are compared before and after treatments in the three experimental groups. Values are mean ± SEM (n = 20, 12 and 14 in Group IIa, IIb, and IIc, respectively) *p<0.05 vs. “Before Treatment”.

In the “NOS-blocked” Groups IIa., IIb. and IIc. iv. administration of L-NAME had no significant effect on acid base or blood gas parameters but increased systemic blood pressure and decreased heart rate (Figure 1.). These changes developed within 25 minutes after L-NAME and remained unaltered later even after the intravenous administration of 1 µg/kg U-46619 (in Group Ia.), 1 ml/kg saline (in Group IIb.) or 10 mg/kg ozagrel (in Group IIc.) (data not shown). The CoBF reduced by more than 25% within the first 25 min after L-NAME administration (Figure 1.) but did not change further until the completion of the experiments in any of these experimental groups (data not shown). Low frequency CoBF oscillations, which were absent under resting conditions, developed after the administration of L-NAME with a dominant frequency of 148±2 mHz and peak magnitude of 5.6±0.5 AU (n = 46). U-46619 significantly increased while ozagrel decreased the magnitude of these oscillations without changing the dominant frequency (Figure 2.). In contrast, saline, the vehicle of U-46619 and ozagrel, failed to induce any changes in the magnitude or frequency of CoBF oscillations (Figure 2.).

*In vitro* experiments in isolated MCAs showed similar results to the *in vivo* observations. In control vessels administration of saline had no effect on the mean vascular tension while subsequent administration of 100 nM U-46619 induced weak (10.9±5.3%) vasoconstriction. In these vessels no vasomotion could be detected before or after saline or U-46619, indicating that U-46619 in this concentration fails to induce any vasomotional activity under physiological conditions (data not shown).

**Figure 3:**
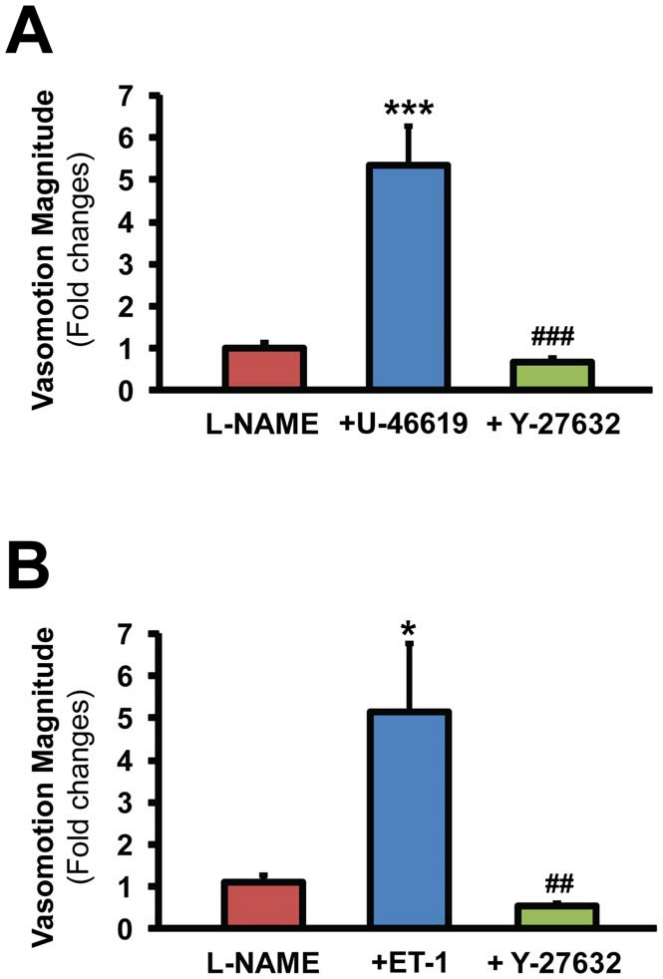
Activation of thromboxane or endothelin receptors induce Rho-kinase dependent vasomotion in NO synthase blocked MCAs. Quantitative analysis of slow wave oscillations with discrete Fourier transformation in L-NAME treated vessels before and after the administration of the TP-receptor agonist U-46619 (Panel A) or endothelin-1 (Panel B) followed by the Rho-kinase inhibitor Y-27632. Values are mean ± SEM fold changes of the peak magnitudes of the power spectra compared to the baseline. *p<0.05, ***p<0.001 vs. L-NAME, ##<0.01 vs. ET-1 and ###<0.001 vs. U-46619 (n = 10–20).

Inhibition of the NO synthesis by L-NAME slightly increased the vascular tone (by 9.5±2.2%) but did not induce vasomotion, except in 3 vessels out of 32 in which the frequency of tension oscillations was 67.2±15.3 mHz. Subsequent stimulation of the TP-receptors with 100 nM U-46619 induced significantly (P<0.001) stronger elevation of the mean vascular tone (by 77.2±6.2%) as compared to the control vessels. Furthermore, U-46619 which failed to induce any vasomotional activity in control vessels, induced strong vasomotion in 16 out of 19 NOS-blocked MCAs with a dominant frequency of 56.1±4.7 mHz. (The lower frequency of these oscillations compared to those of the CoBF in vivo is probably due to the larger diameter of MCAs as compared to cerebral arterioles the resistance of which determines CoBF, since it has been shown that the frequency of vasomotion is negatively related to vessel diameter [27].) Quantitative analysis of the peak magnitude of the Fourier spectra showed a 5.35-fold increase after administration of U-46619 (Figure 3.). In 10 NOS-blocked vessels showing strong vasomotion after U-46619, 10 µM Y-27632 was applied in order to investigate the involvement of Rho-kinase in the mediation of the vascular responses. In all of these vessels Y-27632 abolished the vasoconstriction and vasomotion (Figure 3.).

As mentioned earlier the mean vascular tension after L-NAME+U-46619 treatment was significantly higher as compared to the tension after saline+U-46619 (see above). Therefore, it could be possible that the vasomotion-inducing effect of U-46619 in the former vessels was due to the higher vascular tension and not due to the absence of NO. In order to test this hypothesis in 13 control vessels the vascular tone was increased by 25 mM K^+^ prior to administration 100 nM U-46619. No vasomotion has been observed in any of these vessels athough their mean tension (62.3±8.1%) was similar to that of the L-NAME+U-46619 treated ones.

The above described results indicated that weak pharmacological stimulation of TP-receptors, which induced negligible effects under physiological conditions, evoked vasomotion and CoBF oscillations in NO-deficiency. However, CoBF oscillation which developed after inhibition of NO synthesis could not be reversed completely by inhibition of TXA_2_ synthesis, raising the possibility that other mediators may also be involved in this process. Therefore, in further experiments we aimed to analyze if endothelin-1, which reportedly also activates the Rho-kinase signaling pathway in the cerebrovascular smooth muscle [28], may also induce vasomotional activity in NOS-blocked MCAs. In 10 out of 13 L-NAME pretreated vessels 10 nM endothelin-1 induced strong vasomotion with a dominant frequency of 48.8±7.3 mHz and it was reversible by Y-27632. Quantitatively, the magnitude of the Fourier spectra showed a more than 5-fold increase after administration of endothelin-1 which effect was completely reversed by Y-27632 (Figure 3.). In contrast, administration of 10 nM (or even 100 nM) endothelin-1 to 9 control vessels failed to induce any vasomotional activity (data not shown).

## Discussion

Our results indicate that activation of TP-receptors increases cerebral vasomotion and CBF oscillations in NO-deficiency. This observation is particularly interesting in the light of the high prevalence of pathologic conditions associated with diminished release and/or biological effectiveness of NO, such as atherosclerosis, diabetes, hypertension, ischemia/reperfusion and SAH. Since NO suppresses platelet aggregation as well as vascular release of TXA_2_, NO-deficiency is often associated with activation of TP-receptors. Therefore, experimental conditions of the present study mimicked features of several cerebrovascular diseases. Vasomotion is often enhanced in these pathophysiological states although it is still a question of debate whether CBF oscillations represent the last attempt of the cerebral circulation to prevent neuronal hypoxia or it is already the first sign of the already disrupted regulation. Both of these interpretations can be supported by the observations that CoBF oscillations precede the onset of vasospasm in SAH [11].

NO reportedly suppresses the synthesis of TXA_2_ [29] and inhibition of NOS was recently reported to enhance TXA_2_ release also from cerebrovascular endothelial cells [17]. Our observation that ozagrel inhibited L-NAME induced CoBF oscillations clearly indicates that endogenous TXA_2_ significantly contributes to the development of cerebral vasomotion in NO-deficiency. On the other hand, the cerebrovascular endothelium is not likely to be the only source of TXA_2_ release since L-NAME induced vasomotion in only 3 out of 32 isolated MCAs in vitro, suggesting that vascular TXA_2_ production by itself may not be sufficient to evoke vasomotion even in case of diminished NO synthesis. However, stimulation of the TP-receptors by 100 nM U-46619, which was without any effect under physiological conditions, induced strong vasomotion in the absence of NO indicating a hypersensitivity of the vessels to TXA_2_. Since this effect developed at a constant level of TP-receptor stimulation fluctuations of the cerebrovascular tone and CoBF appear to be rather potentiated but not directly induced by TP-receptor mediated mechanisms.

In a recent study we have shown that both the G_q/11_ and the G_12/13_ heterotrimeric G proteins are involved in the mediation of TXA_2_-induced vasoconstriction [30]. However, the vascular effects of weak TP-receptor stimulation, like those applied in the present study, are primarily induced by the G_12/13_-mediated activation of the small G protein RhoA and Rho-kinase (Németh and Benyó, unpublished observations), the main signaling pathway of calcium-sensitization. Since vasomotion is induced by calcium waves in the smooth muscle [31], TP-receptor mediated calcium-sensitization can enhance the resultant changes of the vascular tension, which is the most plausible explanation of our findings.

Why is this TP-receptor mediated mechanism enhanced in NO-deficiency? As noted above, the increased release of endogenous TXA_2_ can only partly explain our observations. In the cerebral circulation constitutive NO-synthesis maintains a basal vasodilator tone, partly by inhibition of the vasoconstrictor TXA_2_-pathway [16]. TP-receptors are targets of cyclic GMP-dependent kinase (PKG) which induce desensitization by phosphorylation at Ser^331^ of the C-tail domain of the receptor [32]–[33]. Furthermore, NO also interferes with the signaling pathway coupling TP-receptor activation to vascular smooth muscle contraction. For instance, NO reportedly inhibits calcium sensitization by PKG-mediated inhibition of RhoA [34]–[35] and telokin [36], as well as by inhibition of RhoA activation through protein kinase A (PKA)-dependent phosphorylation of Gα_13_ [37]. PKG can also inhibit calcium sensitization by phosphorylating directly the myosin phosphatase targeting subunit (MYPT1) at Ser^695^, resulting in the reduction of phosphorylation at the adjacent inhibitory Thr^696^ site [38]–[40], the target of Rho-kinase [41]. Therefore, in the absence of NO not only the TP-receptors but also these downstream signaling pathways will be released from the tonic inhibitory influence of NO resulting in a sensitized smooth muscle contractile machinery to fluctuations induced by calcium waves. Indeed, in our present study, inhibition of Rho-kinase by Y-27632 completely reversed the vasomotion induced by U-46619 in NO synthase blocked MCAs.

Activation of the RhoA – Rho-kinase pathway has been recently implicated as a key signaling mechanism in the development of several cerebrovascular disorders [42]. TXA_2_ is one but not the only activator of this signaling pathway in the cerebrovascular smooth muscle which conclusion is also supported by our observations that inhibition of the thromboxane-synthesis could only partially reverse the CoBF oscillations after NO synthase blockade while Y-27632 completely abolished vasomotion in vitro. It has been recently demonstrated that endothelin-1, already at low concentrations, activates the RhoA – Rho-kinase pathway in the cerebrovascular smooth muscle [28]. Although we didn't provide direct evidence for its involvement in the generation of CoBF oscillations in vivo, our in vitro data that in NO-deficiency endothelin-1 induces Y-27632 reversible vasomotion supports this hypothesis.

In conclusion, our results indicate that both endogenous TXA_2_ production and hypersensitivity of the TP-receptors and/or the Rho-kinase signaling pathway in the cerebrovascular smooth muscle contribute to the development of CBF oscillations after NO synthase blockade. Pharmacological inhibition of this enhanced reactivity may be beneficial to prevent vasospasm in cerebrovascular disorders associated with NO-deficiency.
